# Anoctamin 1 (Ano1) is required for glucose-induced membrane potential oscillations and insulin secretion by murine β-cells

**DOI:** 10.1007/s00424-015-1758-5

**Published:** 2015-11-18

**Authors:** Raphaël Crutzen, Myrna Virreira, Nicolas Markadieu, Vadim Shlyonsky, Abdullah Sener, Willy J. Malaisse, Renaud Beauwens, Alain Boom, Philippe E. Golstein

**Affiliations:** Laboratory of Cell and Molecular Physiology, Université Libre de Bruxelles, Campus Erasme, Brussels, Belgium; Department of Biochemistry, Université Libre de Bruxelles, Campus Erasme, Brussels, Belgium; Laboratory of Physiology and Pharmacology, Université Libre de Bruxelles, Campus Erasme, Brussels, Belgium; Laboratory of Histology, Histopathology and Neuroanatomy, Université Libre de Bruxelles, Campus Erasme, Brussels, Belgium

**Keywords:** Chloride, TMEM16A, Islet

## Abstract

Anions such as Cl^−^ and HCO_3_^−^ are well known to play an important role in glucose-stimulated insulin secretion (GSIS). In this study, we demonstrate that glucose-induced Cl^−^ efflux from β-cells is mediated by the Ca^2+^-activated Cl^−^ channel anoctamin 1 (Ano1). Ano1 expression in rat β-cells is demonstrated by reverse transcriptase–polymerase chain reaction, western blotting, and immunohistochemistry. Typical Ano1 currents are observed in whole-cell and inside-out patches in the presence of intracellular Ca^++^: at 1 μM, the Cl^−^ current is outwardly rectifying, and at 2 μM, it becomes almost linear. The relative permeabilities of monovalent anions are NO_3_^−^ (1.83 ± 0.10) > Br^−^ (1.42 ± 0.07) > Cl^−^ (1.0). A linear single-channel current–voltage relationship shows a conductance of 8.37 pS. These currents are nearly abolished by blocking Ano1 antibodies or by the inhibitors 2-(5-ethyl-4-hydroxy-6-methylpyrimidin-2-ylthio)-*N*-(4-(4-methoxyphenyl)thiazol-2-yl)acetamide (T-AO1) and tannic acid (TA). These inhibitors induce a strong decrease of 16.7-mM glucose-stimulated action potential rate (at least 87 % on dispersed cells) and a partial membrane repolarization with T-AO1. They abolish or strongly inhibit the GSIS increment at 8.3 mM and at 16.7 mM glucose. Blocking Ano1 antibodies also abolish the 16.7-mM GSIS increment. Combined treatment with bumetanide and acetazolamide in low Cl^−^ and HCO_3_^−^ media provokes a 65 % reduction in action potential (AP) amplitude and a 15-mV AP peak repolarization. Although the mechanism triggering Ano1 opening remains to be established, the present data demonstrate that Ano1 is required to sustain glucose-stimulated membrane potential oscillations and insulin secretion.

## Introduction

The β-cell is known to be an excitable cell for more than 40 years [15–17]. Glucose induces cyclic variations in the membrane potential which are critical for sustained insulin release. Yet, these variations cannot be all ascribed to the specific gating of well-defined ion channels unlike what is established for the membrane potential cyclic variations in the heart [4], as reflected in electrocardiogram. Insulin release requires an increase of the cytosolic calcium concentration ([Ca^2+^]_i_) by any means, i.e., either from the extracellular medium or from intracellular stores. When extracellular glucose concentration is raised (6–10 mM), [Ca^2+^]_i_ increase mainly results from the opening of the plasma membrane L-type Ca^2+^ channels which appears as bursts of spikes superimposed on repetitive slow waves of depolarization, also called active phase. When glucose concentration is further raised, the active phase is progressively extended and finally appears uninterrupted at 25-mM stimulation [56, 81]. The earliest electrical event induced by glucose stimulation is the closure of the K^+^_ATP_ channel that induces membrane depolarization, the latter being reproduced by sulfonylurea drugs [28]. Glucose and sulfonylurea drugs induce long-term fluctuations of the membrane potential with a spiking activity as soon as the threshold of the L-type Ca^2+^ channels is reached, hence increase intracellular Ca^2+^ and insulin release which lasts as long as the stimulus is applied (at least for 1 h) [29, 44, 51, 52, 62]. In sharp contrast, depolarization induced by raising the external potassium concentration results in a single sustained change in membrane potential (and [Ca^2+^]_i_) which induces only a short burst (±7 min) of insulin release, i.e., the so-called “first phase” of insulin secretion with progressive decrease toward basal insulin release [6, 32, 51, 52].

It is however well recognized that closure of K^+^_ATP_ channels alone is not sufficient to reach the threshold of Ca^2+^ channel activation. As β-cells are quite rich in Cl^−^ [9, 26], as early as 1978, Sehlin [66] questioned whether this depolarization could be caused by a Cl^−^ efflux as he demonstrated that glucose induces an increase in ^36^Cl^−^ efflux from ^36^Cl^−^ prelabeled mice islets. Also, severe reduction in anion efflux by incubation in low Cl^−^ (or bicarbonate) media inhibited the electrical bursting pattern together with the ^45^Ca^2+^ uptake, hence the glucose-stimulated insulin secretion (GSIS) [65, 67]. Furthermore, Malaisse et al. [43] established that raising extracellular glucose concentration in the range of 5–20 mM increased proportionally the ^36^Cl^−^ efflux from prelabeled rat islets, thus suggesting that glucose induces the opening of some anion channel [43]. In the present study, we therefore investigated whether such chloride efflux could be mediated by anoctamin 1 (Ano1) channel, also called TMEM16A. Ano1 is a Ca^2+^-activated Cl^−^ channel (CaCC) which belongs to the “anoctamin” family of anion channels recently discovered and cloned (for review, see Pedemonte and Galietta [55] and Kunzelmann [38]). This new family of Cl^−^ channel which shares no sequence similarity with any other membrane protein appears expressed in almost every tissue. In particular, Ano1 is expressed in many different secretory epithelia including the digestive tract: salivary glands, exocrine pancreas, hepatocytes, ileum, and large intestine. It also plays a major role in the pacemaker motility function of the interstitial cells of Cajal [35, 79]. Furthermore, it has also been implicated in cell migration and cancer proliferation [75]. Ano1 knockout mice die soon after birth due to tracheomalacia and also multiple organ impairment [63].

In support to our hypothesis implicating Ano1 in the depolarization critical to open Ca^2+^ channels, Ano1 haplo-insufficient mice exhibit impaired insulin secretion [76], and the present study provides evidence that the opening of the calcium-sensitive Ano1 channel plays a prominent role in glucose-induced membrane potential fluctuations and is therefore critical to achieve a sustained insulin secretion.

## Materials

### Chemicals

Ano1 (TMEM16A) inhibitors T16Ainh-AO1 or 2-(5-ethyl-4-hydroxy-6-methylpyrimidin-2-ylthio)-*N*-(4-(4-methoxyphenyl)thiazol-2-yl)acetamide (T-AO1) and tannic acid (TA) were respectively purchased from Merck Biosciences (Darmstadt, Germany) and Sigma (St Louis, MO, USA). Bumetanide (NKCC1/2 inhibitor, #B-3023), acetazolamide (carbonic anhydrase inhibitor, #A-6011), and poly-l-lysine hydrobromide (#P6282) were purchased from Sigma (St Louis, MO, USA). Compounds of the different buffers were purchased from Merck (Darmstadt, Germany). Collagenase P (#11 213 873 001) and protease inhibitors (complete mini #11 836 153 001) were purchased from Roche (Mannheim, Germany).

### Antibodies

Rabbit polyclonal Ano1 antibodies (ab84115) for western blotting purpose, rabbit polyclonal blocking Ano1 antibodies (ab72984, directed against the fifth and sixth transmembrane domains that constitute the pore-forming region) for patch-clamp and insulin release experiments, and human Ano1 synthetic peptide (ab97423) for immunofluorescence purpose were purchased from Abcam (Cambridge, UK). Goat polyclonal Ano1 antibodies (sc-69343) for immunofluorescence purpose were purchased from Santa Cruz Biotechnology (Dallas, TX, USA). Rabbit anti-goat biotinylated antibody (BA-5000) was purchased from Vector Laboratories (Peterborough, United Kingdom). Horseradish peroxidase (HRP)-conjugated anti-rabbit was purchased from Dako (Glostrup, Denmark).

### Tissue culture and tissue samples

Tissue culture materials were purchased from Sarstedt (Nümbrecht, Germany). Culture media were purchased from Invitrogen Life Technologies (Carlsbad, CA, USA). Experiments were carried out on murine pancreatic islets or dispersed β-cells from Wistar rat and C57BL/6 mice. Animals were anesthetized with Nembutal before decapitation. All experimental procedures were conducted in agreement with the protocol approved by the Ethical and Animal Welfare Committee of the Université Libre de Bruxelles (protocol number 375N). Human cadaveric islet total RNA was kindly provided by Dr Ramon Gomis from the Barcelona Hospital Clinic. Approval of the experimental use of the islets was granted by the Barcelona Hospital ethics committee, and informed consent was given by the donors’ families.

## Methods

### Preparation of rat and mouse pancreatic islets

Pancreatic islets were isolated by collagenase technique [42]. Bile duct was clamped at its entrance in the duodenum, and a cannula was inserted to inject 15-ml Hank’s-glucose solution containing 5.8 mg collagenase P by rat or 2-ml Hank’s–glucose solution containing 4 mg collagenase P by mouse, in order to distend the pancreas and digest the exocrine gland. Hank’s–glucose solution contained the following (in mM): 137 NaCl, 5.4 KCl, 1.2 CaCl_2_, 0.8 MgSO_4_, 0.3 Na_2_HPO_4_, 0.4 KH_2_PO_4_, 4.2 NaHCO_3_, and 11.1 glucose, pH 7.4. The inflated pancreas was extracted and incubated for 15 min (rat) or 9 min (mouse) at 37 °C in a 50-ml conical tube. Digestion was then stopped by addition of 20-ml ice cold Hank’s–glucose solution. The suspension was first gently agitated for 1 min and then vigorously for few seconds. The preparation was transferred in a crystallizing dish before three successive dilution–resuspension–sedimentation–aspiration cycles with 60-ml ice cold Hank’s–glucose solution in order to remove collagenase and floating exocrine acini. Islets were individually collected under a microscope using a pipette and laid at room temperature, on a Petri dish with 4-ml culture medium containing 5 mM glucose (RPMI #21875-034 GIBCO containing 11.1 mM glucose diluted with RPMI #11879-020 GIBCO glucose-free, 10 % heat inactivated fetal bovine serum #10500-064 GIBCO, 50 IU/ml penicillin, 50 μg/ml streptomycin #15070-063 GIBCO). This last step was repeated three to four times to wash islets. Four hundred to six hundred pancreatic islets were obtained from one single rat pancreas, while 20 to 100 islets were isolated from one mouse. The Petri dish containing the islets was then placed at 37 °C in a humidified incubator gassed with 5 % CO_2_ in air. Each preparation was obtained by pooling islets from two animals. The islets were immediately used for experiments.

### Preparation of dispersed rat or mouse islet cells

Freshly isolated pancreatic islets from two animals were suspended in a 1.5-ml tube. The culture medium was discarded and replaced by a 0.5-ml isotonic 0.05 % trypsin–EDTA solution (#25300-054 GIBCO) before incubation at 37 °C, in a humidified incubator gassed with 5 % CO_2_ in air for 9 min. After incubation, the trypsin–EDTA solution was replaced by 0.62-ml culture medium containing 11.1 mM glucose and cells were separated by tenfold gentle suction-pushing with a pipette. Thirty-five to 50 μl of cell suspension was laid onto 1.0-cm-diameter glass poly-l-lysine (PLL)-precoated coverslips (Merck, Darmstadt, Germany). The coating procedure consisted to bath the coverslips for 5 min in a 0.1 or 0.3 mg/ml PLL solution in sterile Milli-Q water before successively rinsing again in sterile Milli-Q water and drying overnight at room temperature in six-well plates. Afterward, cells were placed at 37 °C in a humidified incubator gassed with 5 % CO_2_ in air for 30 min to promote adherence before gentle addition of 4-ml culture medium containing 5 mM glucose. Finally, cells were stored overnight in the incubator before experiments.

### Insulin release

Groups of eight rat pancreatic islets were placed in 1.5- or 2-ml assay tubes. Islets were first preincubated for 30 min in 1-ml Hepes-buffered NaCl solution without bicarbonate or in Hepes and bicarbonate-buffered NaCl solution both supplemented with 1 or 5 mg/ml bovine serum albumin (BSA) and 2.8 mM glucose in presence (or not) of T-AO1, TA, DMSO, rabbit blocking Ano1 antibodies (ab72984, Abcam), or rabbit whole serum and next incubated for 90 min in the same medium at various glucose concentrations: 2.8, 8.3, and 16.7 mM. Hepes-buffered NaCl solution without bicarbonate contained the following (in mM): 140 NaCl, 10 Hepes, 4 KCl, 1 MgCl_2_, and 1 CaCl_2_, pH 7.3, with NaOH. Hepes and bicarbonate-buffered NaCl solution contained the following (in mM): 111 NaCl, 10 Hepes, 5 KCl, 1 MgCl_2_, 1 CaCl_2_, and 24 NaHCO_3_, pH 7.4, with NaOH. After 90 min, 0.2- or 0.5-ml incubation medium was collected. Sampled incubation media were frozen for insulin content determination. Experiments were performed at 37 °C. In presence of bicarbonate media, preincubation and incubation were performed in a humidified incubator gassed with 5 % CO_2_ in air. Insulin content of each sample was measured by radio immmunoassay technique [40] or by ELISA (#10-1250-10 Insulin, rat ELISA, Mercodia, Uppsala, Sweden).

### Patch-clamp experiments

Mouse islets were immobilized at the end of a negatively pressured large borosilicate glass pipette, in the patch-clamp chamber using a micromanipulator (Prior, England). Whole islets were blind-patched between day 0 and day +2. These experiments were carried out on mouse islets, as the sealing could not be maintained for prolonged periods with β-cell from whole rat islet. Dispersed rat or mouse islet cells cultured onto poly-l-lysine-precoated glass coverslips were used between day +1 and day +3. β-Cells (representing 80–90 % of the cells) were visually identified by their large size and their granular appearance [8]. Islets or coverslips were placed in the patch-clamp chamber and continuously perfused (1 ml/min). The temperature in the chamber was maintained at 34 °C with a homemade heater (gift from Prof D. Gall, Department of Biophysics, ULB, Belgium). The patch pipettes were double-step pulled from borosilicate glass capillaries (Hilgenberg GmbH, Malsfeld, Germany) using a vertical puller (PC-10, Narishige International, London, UK). Liquid junction potentials were balanced after formation of a gigaseal contact. Voltages and currents were monitored with a PC-501A amplifier (Warner Instruments, Hamden, CT, USA) or an Axopatch 200A (Axon instruments, Inc., Foster City, CA, USA). Zero-current whole-cell voltages and currents were continuously recorded using WinEDR or WinWCP software (John Dempster, Strathclyde Institute of Pharmacy and Biomedical Sciences, UK). β-Cells were functionally identified by the presence of action potential (AP) bursts induced by stimulating glucose concentration.

#### Voltage measurements

Nystatin-perforated whole-cell configuration patch-clamp experiments were conducted as previously described [70]. Bath solution was a Hepes-buffered NaCl solution without bicarbonate (see “Insulin release” section) supplemented with 2.8 or 16.7 mM d-glucose or low chloride (20 mM) by replacement of 140 mM NaCl in Hepes-buffered NaCl solution without bicarbonate by 12 mM NaCl and 128 mM Na-gluconate, osmolarity 311 or 325 mOsmol/l. Pipette solution contained the following (in mM): 10 NaCl, 20 KCl, 75 K_2_SO_4_, and 10 Hepes, pH 7.2, with KOH, supplemented with 50 to 300 μg/ml nystatin, osmolarity 299 mOsmol/l. Stock solution of nystatin (10 to 30 mg/ml in DMSO) was daily prepared. Filled pipettes had resistances of 6–10 MΩ. Whole-cell configuration induced by nystatin permeabilization was achieved within 10 to 15 min. Leak resistance after sealing was usually at least 3 GΩ. Access resistance (Rs) was <35 MΩ. Cells were stored in incubator and used within 60-min incubation. The effects of T-AO1 or TA on islets were evaluated during active phase after 5-min exposure for at least 3 min or more (30–40 min). APs were counted for 3 min during active phase, 1 min at the beginning, 1 in the center, and 1 at the end. On dispersed cells, the effect of 16.7 mM glucose was evaluated during the spiking activity for at least 30 s or more (1–2 min). The effects of T-AO1 and TA on the potential and APs were evaluated after 1- or 2-min exposure for at least 1 min or more (3–6 min). In low-chloride solution in presence of 200 μM bumetanide, the initial effect of glucose (Ini) was evaluated during 20 to 60 s at the beginning of the spiking activity before the rundown of AP peak. Its effect in presence of bumetanide was also evaluated after 5-min glucose stimulation for a period of at least 5 min (G + 5 min). The effect of 5 mM acetazolamide on glucose and bumetanide was further evaluated after 5-min acetazolamide incubation and for a period of at least 5 min (Acet + 5 min).

#### Chloride current measurements

Chloride currents from rat dispersed β-cells were measured on inside-out single excised patches and whole cell. Filled pipette resistance was 5 MΩ (Fig. 5) except for single-channel currents, 20 MΩ (Fig. 6). Sealing was first performed in Hepes-buffered NaCl solution without bicarbonate containing 2.8 mM glucose (see “Insulin release” section). On inside-out single excised patches (macropatches, filled pipette resistance 5 MΩ, Fig. 5), the bathing solution was replaced after sealing by 150 mM *N*-methyl d-glucamine chloride (NMDG-Cl), pH 7.3, solution containing CaCl_2_ at the indicated concentration (0 to 2 μM), which flowed during 90 s before excision. After excision, leak resistance was 7–25 GΩ. Measurements were performed after 2-min exposure of the cytoplasmic side of the patch to bath solution. Pipette solution contained also 150 mM NMDG-Cl, pH 7.3, in presence or absence of 10 μM T-AO1, active blocking Ano1 antibodies ab72984 1:100 or boiled (heated at 99 °C for 10 min) blocking Ano1 antibodies ab72984 1:100. In the experiments with T-AO1 or antibodies, sealing was performed 5 min before measurements so that a 5-min preincubation occurred. Current traces recorded (sampling rate, 25 kHz; 2-kHz filter setting) were induced by 1500-ms voltage steps from −100 to +100 mV, spaced 20 mV (holding potential, −70 mV, 200 ms before and after each step). Steady-state current–voltage relationship was constructed from the average Cl^−^ currents measured between ms 1400 and 1500 of voltage stimulation. In voltage step experiments, the stability of Cl^−^ currents at the holding potential (−70 mV) before the 11 voltage steps was indicative of the stability of [Ca^2+^]_i_ and of the absence of apparition of sudden leakage currents. Single-channel Cl^−^ currents were also measured on inside-out excised patches (filled pipette resistance 20 MΩ, Fig. 6). After sealing, the bathing medium was replaced just before excision, by a 150 mM NMDG-Cl, pH 7.3, calcium-free medium. Stimulation of CaCC was performed with the same medium containing 1 μM CaCl_2_. Pipette solution was 150 mM NMDG-Cl, pH 7.3, supplemented with 10 μM glibenclamide and 10 μM nifedipine and contained (or not) 100 μM T-AO1 or TA. Currents were corrected for leakage. Leak resistance after sealing was usually 7 to 15 GΩ. Single-channel records were analyzed using pCLAMP software where all event lists of single-channel records were generated by Fetchan program (Molecular Devices, Sunnyvale, CA). *NPo*, the product of the number of channels in a patch (*N*) by the open probability (*Po*), which reflects the channel activity within a patch, was calculated using the equation

$$ NPo={\displaystyle \sum_{i=1}^Ni\ast {t}_i/T} $$

where *T* is the total recording time, *i* is the number of open channels, *t*_*i*_ is the recording time during which *i* channels were open, and *N* is the apparent number of channels within the determined patch (as the highest observable level). Therefore, *NPo* can be calculated without making any assumption about the total number of channels in a patch or the open probability of single channels. All *NP*o values were calculated for 120 s of recording after 15-s stimulation with 1 μM Ca^2+^. To meet the electrophysiological convention, inward Cl^−^ currents from inside-out patches (pipette to cell) were represented as upward transitions. In whole-cell experiments, pipette solution was 150 mM NMDG-Cl, pH 7.2, containing 0 or 1 μM CaCl_2_, in presence or absence of blocking Ano1 antibodies ab72984 1:100 or boiled blocking Ano1 antibodies ab72984 1:100 (cytosolic side of the cell). After sealing, the bathing solution was replaced by 150 mM NMDG-Cl, pH 7.3. The membrane was ruptured by suction to form a whole cell. Access resistance was about 10 MΩ. Series resistance was 50 % compensated. Leak resistance was 7–25 GΩ. Measurements were performed 6 min after whole-cell formation or from 6 to 12 min with blocking Ano1 antibodies. In the experiments with T-AO1, the bathing solution was replaced 2 min before measurements by 150 mM NMDG-Cl, pH 7.3, containing 10 μM T-AO1 which was thus preincubated for 2 min externally. Current traces recorded (sampling rate, 10 kHz; 2-kHz filter setting) were induced by 400-ms voltage steps from −100 to +100 mV, spaced 20 mV (holding potential, −70 mV, 100 ms before and after each step). Current–voltage relationship was constructed from the average Cl^−^ currents measured between ms 380 and 400 of voltage stimulation. In monovalent anion permeability experiments (also whole-cell experiments), an agar-KCl 3M salt bridge provided the junction between the Ag/AgCl reference electrode and the bath. Pipette solution was 150 mM NMDG-Cl, pH 7.2, containing 1 μM CaCl_2_. The bathing solution (150 mM NMDG-Cl) was replaced by 150 mM NMDG-NO_3_ or NMDG-Br, pH 7.3, which flowed during 1.5 min before a first measurement performed 6 min after whole-cell formation. The bathing solution was then exchanged with 150 mM NMDG-Cl for 1.5 min before a second measurement. Current traces recorded (sampling rate, 25 kHz; 2-kHz filter setting) were induced by a slow voltage ramp: from holding potential (−70 mV, 200 ms), a step to −100 mV, 400 ms was applied followed by a 10-s linear voltage ramp from −100 to +100 mV with thereafter a step to holding potential, −70 mV, 200 ms. Parts of ramps from −40 to +40 mV are represented in Fig. 5j, l.

#### Barium current measurements

In whole-cell experiments, cells were first immersed in Hepes-buffered NaCl medium containing 2.8 mM glucose (see “Insulin release” section). After establishing the whole-cell configuration, the medium was then replaced by a barium solution containing (in mM) 75 BaCl_2_, 10 Hepes, 35 NMDG-gluconate, pH 7.3, with NMDG, osmolarity 310 mOsm/l and supplemented (or not) with 100 μM Ano1 inhibitors (T-AO1 or TA) or 10 μM nifedipine. Pipette solution contained (in mM) 103 NMDG-Cl, 10 Hepes, 3 MgATP, 43 NMDG-gluconate, 10^−4^ CaGluconate_2_, pH 7.15 with NMDG, osmolarity 310 mOsm/l. Chloride equilibrium potential was −10 mV. Holding potential was −70 mV. Filled pipette resistance was 5 MΩ. Leak resistance was at least 3 GΩ (usually 4 to 7 GΩ) after establishment of the sealing. Access resistance (Rs) was between 10 and 20 MΩ. The currents have been corrected for leakage and capacity currents. Series resistance was compensated by 50 %. Ba^2+^currents were observed during 200-ms depolarizations to −10 mV from a holding potential of −70 mV in the absence or presence of T-AO1 (100 μM), TA (100 μM), or nifedipine (10 μM) in the bathing medium and normalized (or not) to the capacitance of each cell. To establish the peak inward current (*I*)–voltage(*V*) relationship, 25-ms depolarizations from −50 to + 30 mV by 10-mV voltage steps were applied from a holding potential of −70 mV. Only the first set of measurements was considered for each cell to avoid a rundown of the currents.

### Reverse transcriptase for Ano1

Total RNA from rat and human tissues (pancreas, kidney) was extracted using the AURUM^™^ total RNA fatty acid and tissue kit (Bio-Rad, Hercules, CA, USA), and then, complementary DNA (cDNA) was prepared using the High-Capacity cDNA Reverse Transcription kit (Applied Biosystems, Waltham, MA, USA). RT-PCR reactions were carried out in 20-μl final volume containing the following: 1 μl of cDNA added to 4 μl of Go Taq Green 5× Buffer, 0.2 mM dNTP, 0.5 μM of each primer, and 0.5 U Go Taq^™^ polymerase (Promega, Madison, WI) using MyCycler^™^ Thermal Cycler (Bio-Rad Laboratories, Hercules, USA). Primers used (GenBank accession no. NM_001107564) for rat Ano1 were as follows: forward 5′-GCAGGCCTGGAGCTGGAACG-3′and reverse 5′-GCTCAGCCACCTTGGGCTGG-3′, and those (GenBank no. NM_018043) for human Ano1: forward 5′-CACAGGCGGCCACGATGAGG-3′ and reverse 5′-GGGGTGGTCCTGCTTGACGC-3′. Thermocycler conditions were:denaturation at 95 °C for 90 s followed by 35 cycles (95 °C for 30 s, 60 °C for 30 s, 72 °C for 1 min) and finished by a final elongation at 72 °C of 5 min. Amplicons were separated on a 1.2 % agarose gel in TAE buffer (40 mM Tris base, 20 mM acetic acid, 1 mM EDTA, pH 7.4, supplemented with 0.5 μg/ml ethidium bromide). Gels were visualized by UV translumination, using a GelDoc apparatus (Fusion FX5 Vilber Lourmat). A sequence analysis was performed on purified PCR products: 5 μl of PCR products was treated with ExoSAP-IT enzyme (Affymetrix Santa Clara, CA, USA) in order to remove excess dNTPs and residual primers. The reaction was carried out at 37 °C for 15 min and stopped by heating at 80 °C for 15 min. Two microliters of the mix PCR/ExoSAP-IT was mixed with 2 μl Ano1 primers 3.5 pmol/μl, and volume was finally completed to 7 μl with water. Sequencing was subcontracted to external services (Beckman coulter genomics, Danvers, MA, USA), and the sequences were compared using nucleotide BLAST search program (BLAST2SEQ) of NCBI database. (http://www.ncbi.nlm.nih.gov).

### Immunodetection analysis of Ano1 in rat islets

Immunodetection of Ano1 was performed by Western blotting. Islets were isolated, as previously described, from two rats and lysed into 60 μl ice cold RIPA lysis buffer (50 mM Tris base, pH 7.4, 150 mM NaCl, 1 % NP-40, 0.25 % sodium desoxycholate, and supplemented with Roche protease inhibitor tablet). Protein concentration, measured by Bradford, was 4 μg/μl. After centrifugation (16,000 rcf, 15 min), samples were supplemented with Laemmli buffer/1 % dithiothreitol (Laemmli 4×: 250 mM Tris–HCl, pH 6.8, 40 % glycerol, 5 % SDS, bromophenol blue; sample: Laemmli buffer 4×, 3 *v*:1 *v*) and denatured by heating at 70 °C for 30 min. Proteins were separated on a 5 % acrylamide gel and transferred to a PVDF membrane. Procedure was as previously described [45]. After blocking, the membrane was successively incubated overnight at 4 °C with a rabbit polyclonal anti-ANO1 (ab84115, Abcam) 1:1000, rinsed, and incubated at room temperature for 2 h with HRP-conjugated anti-rabbit. Detection was performed by exposure to enhanced chemiluminescence (Amersham). Human thyroid extract was used as positive control. Molecular weight of rat Ano1 was estimated at 119 kDa, from *Rattus norvegicus* peptide sequence (NP_001101034.1, NCBI).

### Immunofluorescence detection of Ano1 in rat pancreas sections

Pancreas was quickly dissected and further fixed by overnight immersion in 4 % (*w*/*v*) paraformaldehyde in 0.1 M phosphate buffer, pH 7.4. The tissue was then transferred to successive graded sucrose solutions (10, 20, and 30 %, overnight each) and finally embedded in Tissue-Tek OCT^™^ compound (Sakura Finetek Europe, Leiden, the Netherlands), snap-frozen in cold 2-methylbutane, and stored at −80 °C. Cryosections (10 μm) were cut on a cryostat (Leitz, Iena, Germany), mounted on glass slides (International Medical Products, Brussels, Belgium), coated with 0.1 % poly-l-lysine, and stored at −20 °C until use. Before the immunostaining immunofluorescence procedure, the slides were submitted to an epitope unmasking procedure: the slides were immerged in a 0.01 M citrate buffer, pH 6, and boiled in a microwave oven for 10 min (2450 MHz, 850 W). The slides remained in the plastic jars for 15 min until cooling to room temperature, were then washed with a Tris-buffered saline solution (TBS, composition in mM: 50 Tris–HCl, 110 NaCl, pH 7.5) and dropped for 30 min in methanol containing 0.3 % hydrogen peroxide in order to block endogenous peroxidase activity. Slides were quickly washed in water, preincubated for 60 min in TBS supplemented with normal rabbit serum (NRS) 1:10, and incubated overnight at room temperature with either goat polyclonal anti-Ano1 sc-69343 (Santa-Cruz) 1:50 or for specificity control in a mix of anti-Ano1 sc-69343:Ano1 peptide ab97423 (Abcam) in ratio of 1:8 preincubated for 2 h in TBS containing NRS 1:100. The slides were washed in TBS and incubated for 30 min with a secondary rabbit anti-goat biotinylated antibody BA-5000 (Vector Laboratories) 1:300, rinsed in TBS, and incubated for 1 h with a streptavidin–peroxidase complex. The slides were washed again in TBS and incubated for 10 min with Tyramide Alexa Fluor 488 1:100 in the amplification buffer provided in the kit (T-20932, Thermo Fisher Scientific, Waltham, MA, USA). Finally, after rinsing twice in TBS, the cryostat slides were washed in water and mounted using Glycergel (Dako, Glostrup, Denmark) supplemented with 2.0 % of the antifading compound 1,4-diazobicyclo[2.2.2]octane (DABCO, Sigma, St Louis, MO, USA). Sections were examined with a Zeiss Axioplan microscope, and images were acquired using an AxioCam HRc camera. After the images were captured, a counterstaining of the same slides with hematoxylin and eosin confirmed the localization of the immunofluorescence signal at the level of the pancreatic islets.

### Statistics

Results are presented as mean values (±SEM) with the number of individual experiments (*n*) except for Fig. 6c where all the points are showed. The normality of population distributions was assessed by Shapiro–Wilk tests. Datasets with normal distributions were compared using parametric tests. Datasets containing at least a non-normal distribution were compared using nonparametric tests. Statistic tests were performed using SPSS 22 software (IBM, Armonk, NY).

## Results

### Expression of Ano1 in rat pancreatic islets

**Fig. 1:**
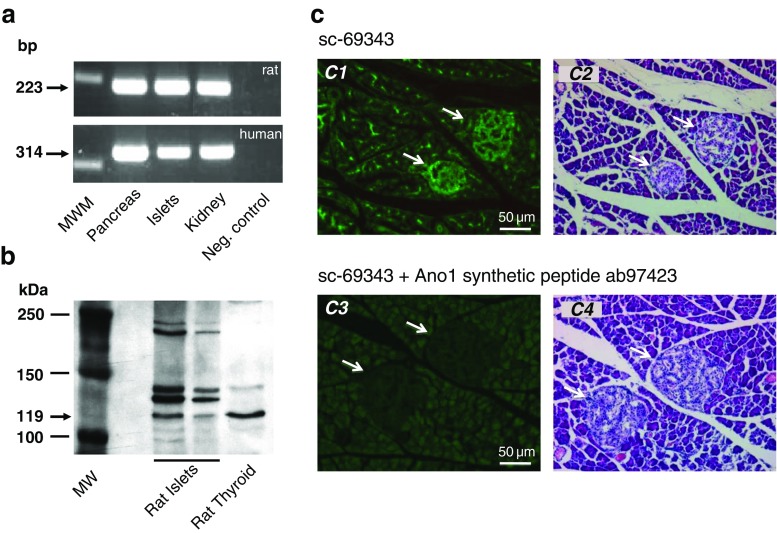
Detection of Ano1 in pancreas and pancreatic islets. **a** RT-PCR of cDNA prepared from mRNA extracted from rat and human tissues. Transcripts of the expected size for Ano1 are observed (rat: 223 bp; human 314 bp). The 300-bp band is shown in the molecular weight marker column (MWM). Positive control: kidney. Negative control (Neg. control): no DNA. The sequencing of PCR products confirmed 100 % identity with the reference sequence for rat Ano1 cDNA complementary of rat Ano1 mRNA. **b** Western blot of Ano1 in rat islets, from *left* to *right*: molecular weight column (MW) showing the 100-, 150-, and 250-kDa bands, 80 μg rat islet lysate, 30 μg rat islet lysate, and 30 μg human thyroid lysate (positive control). Ano1 is detected at 119 kDa. **c** Immunofluorescence staining of pancreas section*. c1* Immunohistochemical labeling (green-fluorescent Tyramide Alexa 488) of Ano1 in a section photomicrograph of rat pancreas. Most of the islet cells and acinar cells (at the level of apical pole) are labeled. *c2* Counterstaining labeling by hematoxylin–eosin performed on the slice used for *c1. c3* Specificity control: immunohistochemical labeling of Ano1 in a section photomicrograph of rat pancreas. The primary goat Ano1 antibodies (sc-69343) were coincubated in the presence of Ano1 synthetic peptide (ab97423) in a ratio 1:8. The labeling disappears. *c4* Counterstaining labeling by hematoxylin–eosin performed on the slice used for *c3. Arrows* show islets. *Scale bar* is 50 μm

By RT-PCR, the transcript of Ano1 was found in total pancreas and in purified islet preparation in both rat and human species. As kidney is well known to express Ano1 [23], messenger RNA (mRNA) extracted from kidney was used as positive control (Fig. 1a). The comparison of sequences of PCR products obtained by amplification of rat total pancreas, isolated islets, and kidney cDNAs vs. reference sequences was performed using BLAST2SEQ program (accession number: NM_001107564.1) and confirmed 100 % identity. The expression of Ano1 protein in whole rat islets was assessed by western blotting (80 and 30 μg prot/lane) using a human thyroid lysate as positive control. This clearly established that Ano1 is expressed in rat islet cells, as observed in human and mice β-cells [27]. Ano1 cellular distribution was further studied in the pancreas by immunofluorescence. A strong Ano1 staining was observed at the level of Langerhans’ islet and in acinar cells at the apical side of the acini (Fig. 1c). Almost all the cells of the islet were labeled (Fig. 1 (c1)) as confirmed by the hematoxylin–eosin counterstaining (Fig. 1 (c2)) which was performed on the same slice. Coincubation of the primary goat Ano1 antibodies with Ano1 synthetic peptide (which competes with tissue Ano1) in a ratio 1:8 completely prevented the labeling (Fig. 1 (c3)).

### Effect of Ano1 on GSIS in rat pancreatic islets

**Fig. 2:**
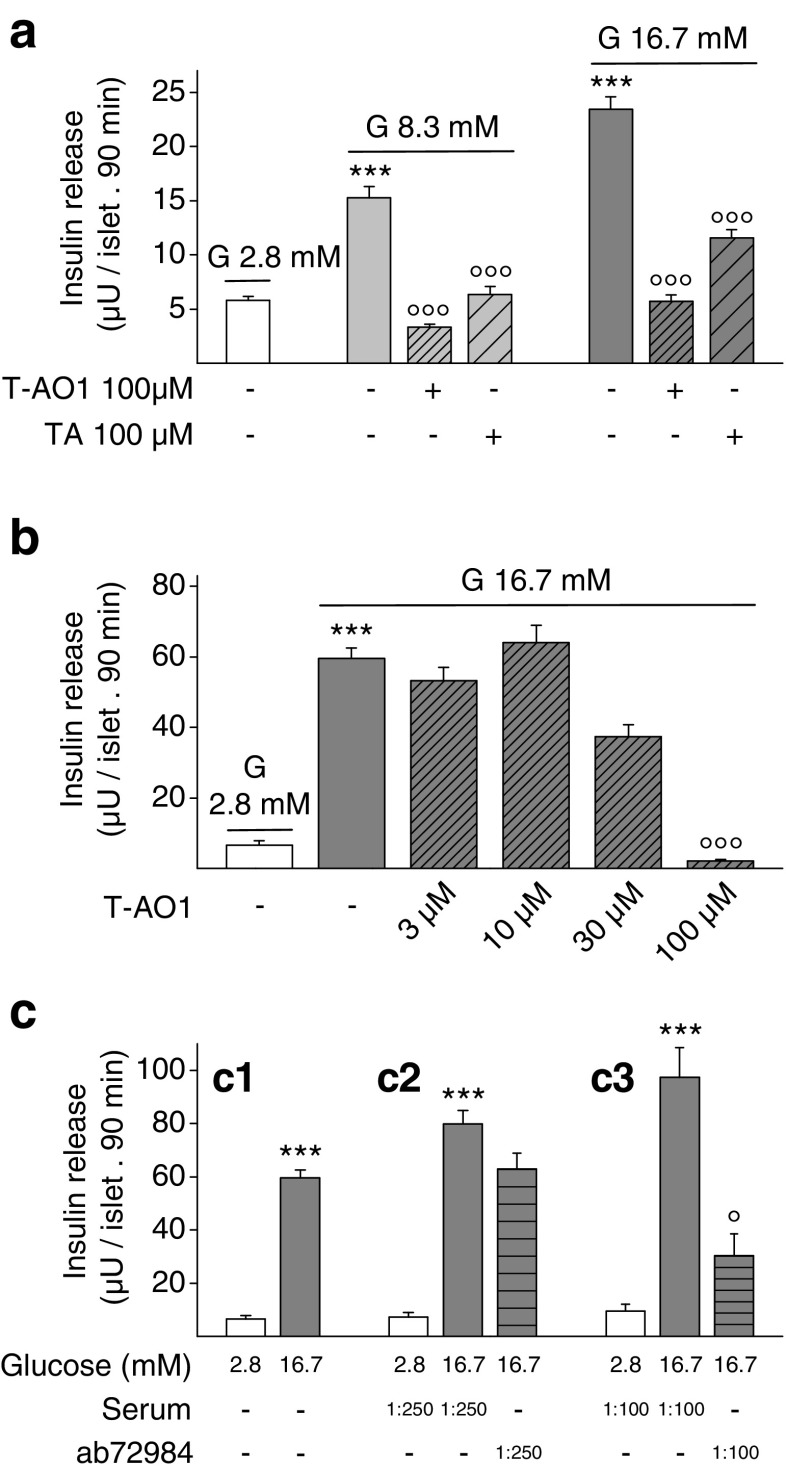
Insulin release from rat pancreatic islets is drastically reduced in the presence of Ano1 inhibitors and blocking Ano1 antibodies. Islets were first preincubated in 2.8 mM glucose, in the presence (or not) of T-AO1, TA, or blocking Ano1 antibodies (ab72984), and then incubated for 90 min in the same medium with different glucose concentrations (2.8, 8.3, and 16.7 mM). The experiments were always performed on groups of eight islets isolated from preparations of two rats. **a** Effect of T-AO1 and TA (100 μM) on glucose-stimulated insulin secretion (GSIS) from rat islets in Hepes-buffered NaCl solution without bicarbonate as used in the patch-clamp voltage measurements (*n* = 58–59 from six preparations in conditions without inhibitors, *n* = 29–30 from three preparations in conditions with inhibitors). Preincubation with T-AO1 or TA completely abolished the increment in insulin output on glucose 8.3-mM stimulation. With 16.7 mM glucose, no increase was observed in the presence of T-AO1, while the relative increase was weak with TA (32.6 ± 10.6 %). **b** Dose-dependent effect of T-AO1 on 16.7 mM GSIS from rat islets in Hepes and bicarbonate-buffered NaCl solution (*n* = 19–20 from three preparations except for 10 μM T-AO1, *n* = 32). T-AO1 100 μM prevented any increase in GSIS. **c** Effect of rabbit blocking Ano1 antibodies ab72984 (in whole serum) on 16.7 mM GSIS from rat islets in Hepes and bicarbonate-buffered NaCl solution. *c1* No antibody/no serum (*n* = 20 from two preparations). *c2* ab72984 or serum 1:250 and *c3* ab72984 or serum 1:100 (*c2* and *c3*, *n* = 9–12 from three preparations). The relative increment in presence of 1:100 antibodies was reduced to 23.9 ± 16.0 % with a secretion value not significantly different from control (*P* = 0.15 vs. 2.8 mM glucose). One-way ANOVA test on **a**, *P* = 1.41 × 10^−46^; Kruskal–Wallis test on **b**, *c2*, *c3*, *P* < 0.001; Mann–Whitney test on *c1*, *P* < 0.001. *** *P* < 0.001 vs. 2.8 mM glucose condition; °*P* < 0.05, °°°*P* < 0.001 vs. 16.7 mM glucose-stimulated condition (Sidak tests in **a**; Mann–Whitney-type tests with Dunn–Bonferroni correction in **b**, *c2*, *c3*)

In Hepes-buffered NaCl solution without bicarbonate (Fig. 2a), 8.3 and 16.7 mM GSIS, respectively, represented 263.2 ± 33.9 (*n* = 58, *P* = 2.79 × 10^−12^) and 404.2 ± 48.5 % (*n* = 59, *P* < 10^−13^) of the basal secretion (*n* = 58). When incubated with 8.3 mM glucose, T-AO1 and TA 100 μM completely prevented the insulin output increase (T-AO1: *n* = 30, *P* = 3.40 × 10^−13^ vs. 8.3 mM glucose, *P* = 0.878 vs. 2.8 mM glucose and TA: *n* = 29, *P* = 1.30 × 10^−7^ vs. 8.3 mM glucose, *P* > 0.999 vs. 2.8 mM glucose). In presence of 16.7 mM glucose, T-AO1 also abolished the relative increment in insulin output (*n* = 30, *P* < 10^−13^ vs. 16.7 mM glucose, *P* > 0.999 vs. 2.8 mM glucose), while TA reduced it to 32.6 ± 10.6 % (*n* = 29, *P* = 6.39 × 10^−13^ vs. 16.7 mM glucose, *P* = 0.00292 vs. 2.8 mM glucose). GSIS (16,7 mM) in the absence of bicarbonate (Fig. 2a) represented 39.3 ± 4.3 % of the insulin release measured in 24-mM bicarbonate medium (Fig. 2b, *P* = 1.16 × 10^−19^ by independent Student’s *t* test), in agreement with the observation reported by Henquin and Lambert [29]. In bicarbonate medium, 16.7 mM GSIS represented 905.7 ± 218.5 % of basal secretion (Fig. 2b, *n* = 20, *P* < 0.001 vs. 2.8 mM glucose). T-AO1 dose-dependently decreased 16.7 mM GSIS (Fig. 2b, *n* = 19–32). No significant inhibitory effect was observed for T-AO1 concentrations up to 30 μM (3 μM, *P* > 0.999; 10 μM, *P* > 0.999; 30 μM, *P* = 0.371 vs. 16.7 mM glucose) although the 16.7-mM GSIS increment in presence of 30 μM T-AO1 represented only 58.1 ± 13.5 %. T-AO1 100 μM prevented any increase in GSIS (*P* < 0.001 vs. 16.7 mM glucose, *P* > 0.999 vs. 2.8 mM glucose). The importance of Ano1 in GSIS was also confirmed by the abolition of 16.7-mM GSIS increment by the blocking Ano1 antibodies ab72984 [14] in bicarbonate medium (Fig. 2c). The relative increment at 1:100 dilution was 23.9 ± 16.0 % (Fig. 2 (c3), *n* = 9–12, *P* = 0.036 vs. 16.7 mM glucose and *P* = 0.15 vs. 2.8 mM glucose).

### Effect of Ano1 inhibitors T-AO1 and TA on the membrane potential of β-cell from whole mouse islet

**Fig. 3:**
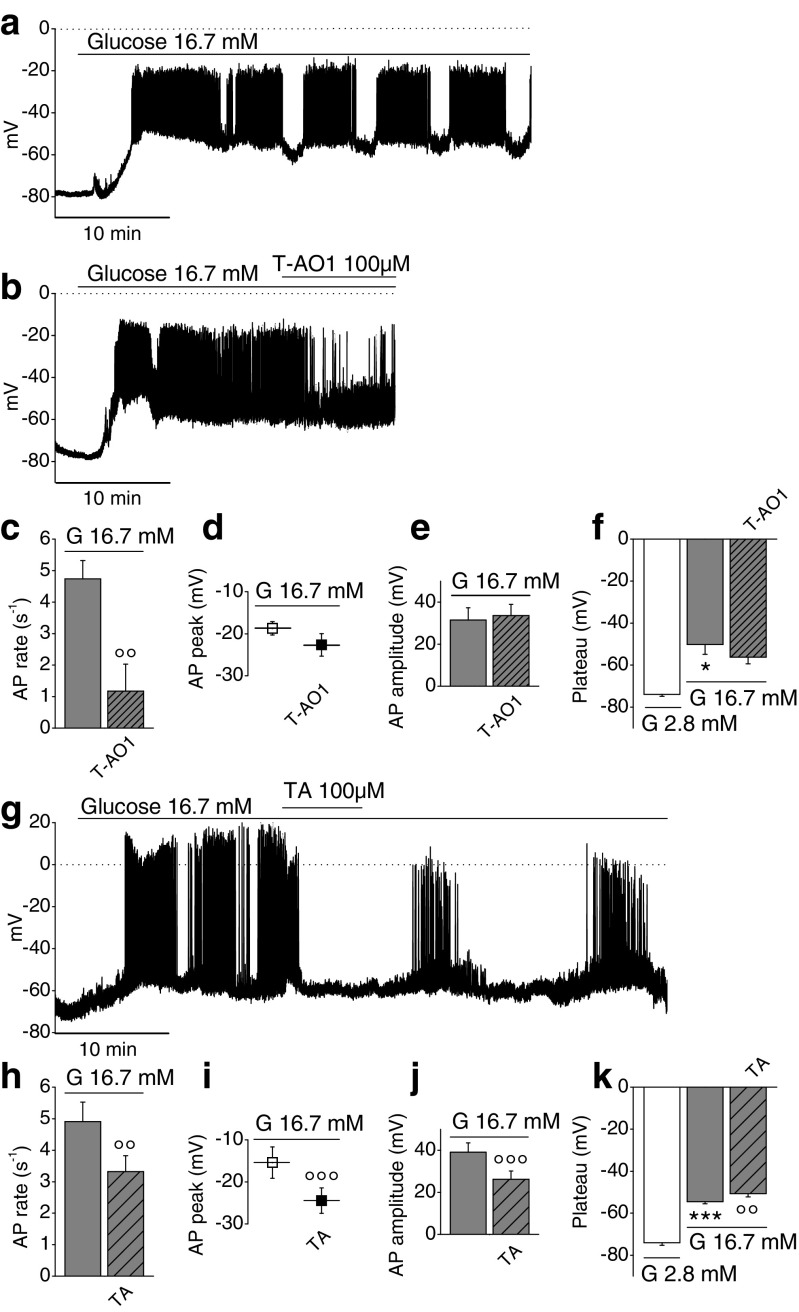
Effect of Ano1 inhibitors on the membrane potential of β-cell from whole mice islet stimulated with 16.7 mM glucose. **a**, **b**, **g** Representative zero-current nystatin-perforated patch-clamp voltage recordings. Sampling rate, 18 kHz; 2-kHz filter setting. *Dotted lines* represent zero-voltage level. **a** Glucose-stimulated cell (16.7 mM glucose). **b** Glucose-stimulated cell ± 100 μM T-AO1 in the bathing medium. **c** Effect of T-AO1 (*n* = 3) on action potential (AP) rate, in presence of glucose. **d** Effect of T-AO1 (*n* = 3) on AP peak, in presence of glucose. **e** Effect of T-AO1 (*n* = 3) on AP amplitude, in presence of glucose. **f** Effect of T-AO1 (*n* = 3) on the membrane plateau potential during active phase in presence of glucose. **g** Glucose-stimulated cell ± 100 μM TA in the bathing medium. **h** Effect of TA (*n* = 7) on action potential rate, in presence of glucose. **i** Effect of TA (*n* = 9) on AP peak, in presence of glucose. **j** Effect of TA (*n* = 9) on AP amplitude, in presence of glucose. **k** Effect of TA (*n* = 9) on the membrane plateau potential during active phase, in presence of glucose. The experiments were performed on ten preparations of mice islets. Friedman test on **f**, *P* = 0.05; repeated measures ANOVA test on **k**, *P* = 6.88 × 10^−10^. *P < 0.05, ***P < 0.001 vs. 2.8-mM glucose condition; °°*P* < 0.01, °°°*P* < 0.001 vs. 16.7-mM glucose condition (paired Student’s *t* tests in **c**–**e**, **h**–**j**; Wilcoxon type tests with Dunn–Bonferroni correction in **f**; least significant difference tests in **k**)

Zero-current nystatin-perforated patch-clamp voltage recordings were performed on cells from intact islets stimulated with 16.7 mM glucose. After a first prolonged depolarization, a cyclic pattern of membrane potential fluctuations was observed with alternating depolarized active phases (slow waves with bursts of spikes) and partially repolarized silent phases, as previously reported [25, 30]. Figure 3a shows such a representative membrane voltage recording in a cell stimulated with 16.7 mM glucose.

The effects of T-AO1 and TA inhibitors (100 μM) were evaluated after 5-min exposure. APs were counted for 3 min during the active phase (1 min at the beginning, 1 in the middle, and 1 at the end). Representative membrane voltage recordings in presence of T-AO1 or TA are presented in Fig. 3b, g. The greatest impact of inhibitors occurred on AP rate: T-AO1 largely reduced glucose-stimulated AP rate, averaging 4.74 ± 0.58 s^−1^ to 1.17 ± 0.86, i.e., by 78.7 ± 14.1 % (Fig. 3c, *n* = 3, *P* = 6.30 × 10^−3^). A reduction from 4.91 ± 0.62 to 3.32 ± 0.50 s^−1^, thus by 33.5 ± 5.1 %, occurred with TA (Fig. 3h, *n* = 7, *P* = 2.44 × 10^−3^). Ano1 inhibition with TA repolarized also the AP peak that averaged −15.41 ± 3.73 to −24.47 ± 3.03 mV, thus by 9.06 ± 1.13 mV (Fig. 3i, *n* = 9, *P* = 4.18 × 10^−5^), but T-AO1 repolarization from −18.71 ± 1.58 to −22.65 ± 2.68 mV, i.e., by 3.94 ± 1.41 mV, was not significant (Fig. 3d, *n* = 3). TA also provoked a significant reduction of 33.9 ± 2.5 % of AP amplitude (39.09 ± 4.40 mV in its absence vs. 26.23 ± 3.89 mV in its presence, Fig. 3i, *n* = 9, *P* = 3.00 × 10^−6^), while T-AO1 did not (Fig. 3e, *n* = 3), Glucose stimulation depolarized the cell (resting potential −74.02 ± 0.93 mV, *n* = 12), and during active phase, the plateau potential averaged −53.42 ± 1.34 mV (*n* = 12, *P* = 5.19 × 10^−8^). The inhibitors did not repolarize the plateau potential: Fig. 3f (T-AO1) and Fig. 3k (TA). The fraction of plateau phase (FOPP, relative duration of the active phase) of 16.7 mM glucose-stimulated β-cell with glucose approached a value of 60 % [56, 81]. In presence of TA, the duration of both silent and active phases was about threefold extended with almost no effect on FOPP which barely decreased from 0.615 ± 0.026 to 0.589 ± 0.059 (*n* = 6, *P* = 0,733, paired *t* test).

### Effect of Ano1 inhibition on the membrane potential from rat and mice dispersed β-cells

**Fig. 4:**
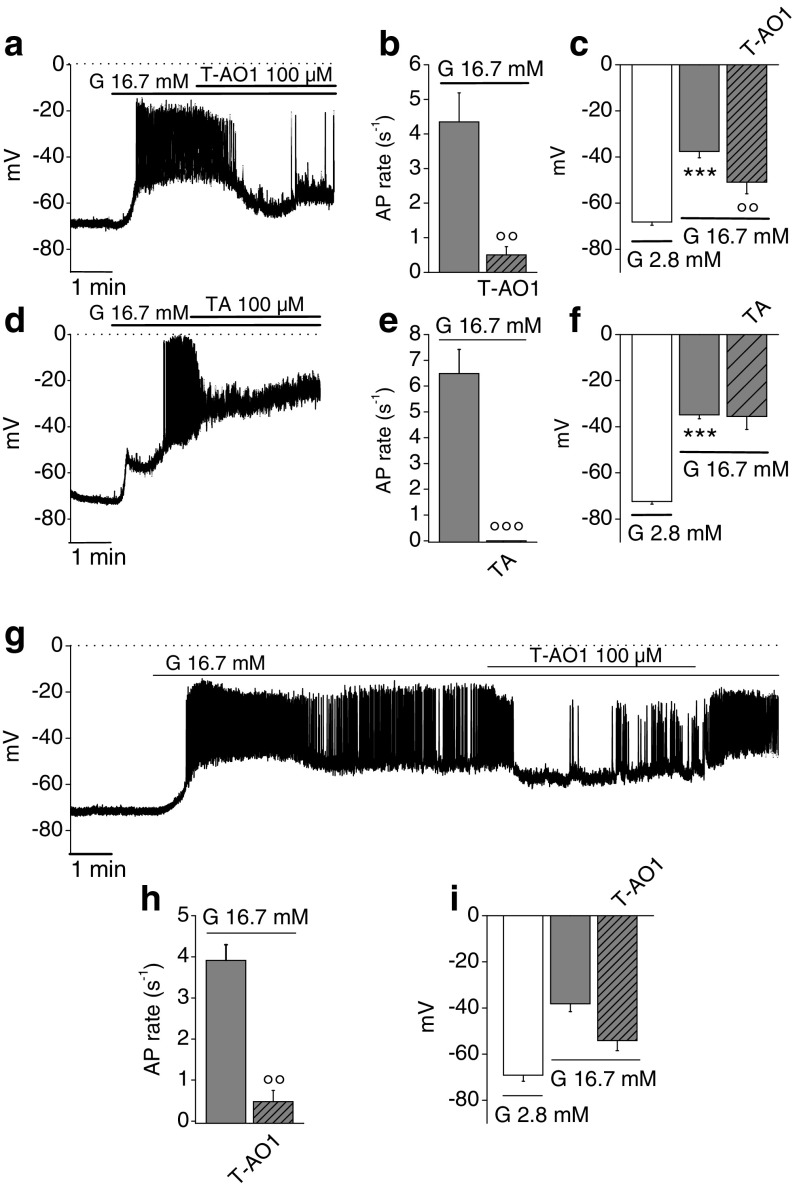
Effect of Ano1 inhibitors on the membrane potential of 16.7 mM glucose-stimulated murine dispersed β-cells. Zero-current nystatin-perforated patch-clamp voltage recordings performed on dispersed β-cells stimulated with 16.7 mM glucose before incubation with T-AO1 or TA (100 μM) in the bathing medium. Sampling rate, 18 kHz; 2-kHz filter setting. *Dotted lines* represent zero-voltage level. **a**–**f** Experiments carried out on rat dispersed β-cells, *n* = 7 from three preparations of rat dispersed islet cells in presence of T-AO1 and *n* = 8 from two preparations of rat dispersed islet cells with TA. **a** Representative recording ± T-AO1. Effect of T-AO1 **b** on AP rate and **c** on average membrane potential. **d** Representative recording ± TA. Effect of TA **e** on AP rate and **f** on average membrane potential. **g**–**i** Experiments carried out on mice dispersed β-cells, *n* = 4 from one preparation of mice dispersed islet cells. **g** Representative recording ± T-AO1. Effect of T-AO1 **h** on AP rate and **i** on average membrane potential. Repeated measures ANOVA test on **c**, *P* = 0.00123; **f**, *P* = 2 × 10^−4^; Friedman test on **i**, *P* = 0.18. ***P < 0.001 vs. 2.8 mM glucose condition; °°*P* < 0.01, °°°*P* < 0.001 vs. 16.7 mM glucose condition (least significant difference tests in **c**, **f**; paired Student’s *t* tests in **b**, **e**, **h**)

Zero-current nystatin-perforated patch-clamp voltage recordings were performed on single dispersed β-cells stimulated with glucose. Only cells showing a resting potential of −70 ± 8 mV were examined: 16.7 mM glucose induced a pattern of electrical activity with numerous repetitive fast-spiking activity. The addition of T-AO1 or TA into the bathing medium is shown in Fig. 4a, d for rat cells and in Fig. 4g for mice cells. Glucose depolarized rat β-cells from an average resting potential of −70.43 ± 1.00 mV to an average potential of −36.12 ± 1.52 mV (*n* = 15, *P* = 3.99 × 10^−11^, paired *t* test). The main modification in the oscillatory pattern in presence of the inhibitors occurred in AP. The AP rate was drastically reduced from 4.35 ± 0.84 to 0.50 ± 0.24 s^−1^, i.e., by 90.3 ± 3.3 % in presence of T-AO1 (Fig. 4b, *n* = 7, *P* = 0.00128), or inhibited by 100 % in presence of TA (Fig. 4e, *n* = 8, *P* = 2,19 × 10^−4^). The average AP rate measured on the stimulated cells was 5.49 ± 0.67 s^−1^ (*n* = 15). T-AO1 partially repolarized the glucose-stimulated cell from a potential of −37.61 ± 2.70 to −50.94 ± 4.97 mV, thus by 13.33 ± 2.58 mV (Fig. 4c, *n* = 7, *P* = 0.00209), while TA did not (Fig. 4f, −34.82 ± 1.65 mV in its absence, −35.48 ± 5.69 mV in its presence; *n* = 8, *P* = 0.907). Similar effects were observed in mice β-cells: glucose (16.7 mM) depolarized the cell from −69.12 ± 2.64 mV (resting potential at 2.8 mM glucose) to −38.17± 3.46 mV (*n* = 4). T-AO1 quite potently and significantly reduced AP rate from 3.91 ± 0.38 to 0.48 ± 0.27 s^−1^, i.e., by 87.4 ± 7.2 % (Fig. 4h, *n* = 4, *P* = 0,00602). T-AO1 repolarized the cell from −38.17 ± 3.47 to −54.10 ± 4.44 mV, thus by 15.94 ± 5.96 mV, although this effect did not reach statistical significance threshold (Fig. 4i, *n* = 4). Interestingly, the washout of T-AO1 inhibitor seemed to reverse its effects (with restored spiking activity), while the effect of TA did not show any recovery during the period of observation (Figs. 3g and 4g).

### Chloride currents from rat β-cells (inside-out excised macropatches and whole cell) display Ano1 properties

**Fig. 5:**
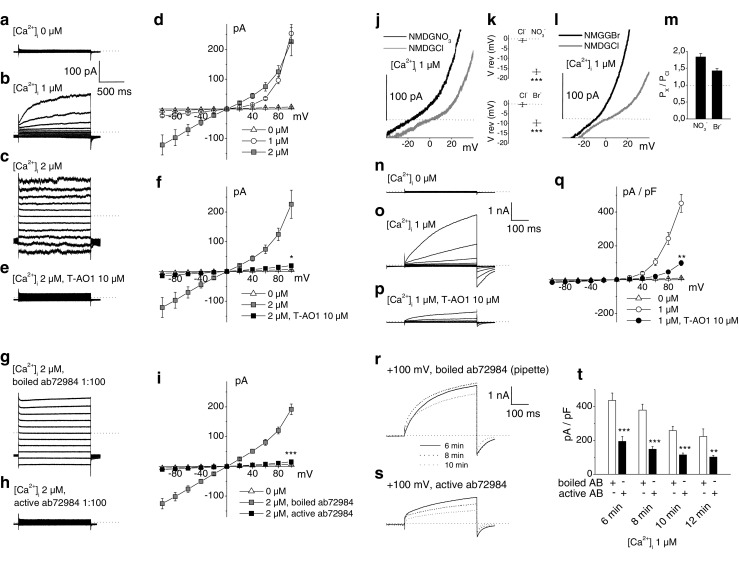
Chloride currents from rat β-cells (inside-out excised macropatches and whole cell). **a–i** Chloride currents from inside-out single excised patches. Pipette and bath solutions contained 150 mM NMDG-Cl. Sampling rate, 25 kHz; 2-kHz filter setting. Filled pipette resistance, 5 MΩ. Current traces recorded were induced by 1500-ms voltage steps from −100 to +100 mV, spaced 20 mV (holding potential, −70 mV, 200 ms before and after each step). *Dotted lines* indicate zero-current level. **a**–**c** Representative recordings of Cl^−^ currents. The cytosolic face was exposed to bath solutions with different [Ca^2+^]: 0 μM in **a**, 1 μM in **b**, and 2 μM in **c. d** Steady-state current–voltage relationships of Cl^−^ currents at 0 μM Ca^2+^ (*n* = 12), 1 μM Ca^2+^ (*n* = 5), and 2 μM Ca^2+^ (*n* = 8). **e** Representative recording of Cl^−^ currents stimulated by 2 μM Ca^2+^ in the presence of T-AO1 10 μM in the pipette. **f** Steady-state current–voltage relationships of Cl^−^ currents at 2 μM Ca^2+^ in the absence (*n* = 8) or presence (*n* = 12) of T-AO1 10 μM in the pipette and at 0 μM Ca^2+^ (*n* = 12). **g**, **h** Representative recordings of Cl^−^ currents stimulated by 2 μM Ca^2+^, respectively, in the presence of boiled or active blocking Ano1 antibodies ab72984 1:100 in the pipette. **i** Steady-state current–voltage relationships of Cl^−^ currents at 2 μM Ca^2+^ in the presence of boiled (*n* = 18) or active (*n* = 13) blocking Ano1 antibodies ab72984 1:100 in the pipette and at 0 μM Ca^2+^ (*n* = 12). **j**–**t** Whole-cell Cl^−^ currents from dispersed β-cells. Pipette and bath solutions contained 150 mM NMDG-Cl except in anion selectivity experiments where the bath Cl^−^ was replaced by NO_3_^−^ or Br^−^ for repeated measures. Sampling rate, 10 or 25 kHz; 2-kHz filter setting. Filled pipette resistance, 5 MΩ. *Dotted lines* indicate zero current or P_x_/P_Cl_ = 1 level. **j**, **l** Representative current traces from β-cells induced by voltage ramps (20 mV/s) at 1 μM Ca^2+^ (pipette). Bath NMDG-Cl solution was replaced by either NMDG-NO_3_ in **j** or NMDG-Br in **l. k** Nitrate and bromide anions shift the reversal potential (V rev) toward negative values (*n* = 11 and 9, respectively). **m** Permeability ratios (P_x_/P_Cl_) of nitrate and bromide anions calculated from the shifts of the reversal potentials in **k** using Goldman, Hodgkin, and Katz equation. **n**–**t** Current traces recorded were induced by 400-ms voltage steps from −100 to +100 mV, spaced 20 mV (holding potential, −70 mV, 100 ms before and after each step). **n**–**p** Representative recordings of whole-cell Cl^−^ currents at 0 μM Ca^2+^ in **n**, 1 μM in **o**, and 1 μM in the presence of T-AO1 10 μM in the bath medium in **p. q** Normalized current–voltage relationships of whole-cell Cl^−^ currents at 1 μM Ca^2+^ in the absence (*n* = 21) or presence (*n* = 15) of T-AO1 10 μM in the bath medium and at 0 μM Ca^2+^ (*n* = 14). **r**, **s** Representative recordings of whole-cell Cl^−^ currents evoked by 1 μM Ca^2+^ at +100 mV after 6-, 8-, and 10-min membrane rupture in the presence of boiled Ano1 antibodies ab72984 1:100 in the pipette (**r**) or active Ano1 antibodies ab72984 1:100 in the pipette (**s**). **t** Normalized whole-cell Cl^−^ currents evoked by 1 μM Ca^2+^ at the end of the +100-mV voltage step in the presence of boiled Ano1 antibodies ab72984 1:100 or active Ano1 antibodies ab72984 1:100 in the pipette after 6 min (*n* = 14 and 13), 8 min (*n* = 12 and 12), 10 min (*n* = 10 and 10), and 12 min (*n* = 5 and 8) membrane rupture. Experiments of Fig. 5 were carried out on six preparations of rat dispersed islet cells. Kruskal–Wallis tests on **f**, **i**, **q**, *P* < 0.001, *P < 0.05, **P < 0.01, ***P < 0.001 vs. control (Mann–Whitney-type tests with Dunn–Bonferroni correction in **f**, **i**, **q**, paired Student’s *t* tests in **k**, independent Student’s *t* tests in **t**)

Figure 5 shows Cl^−^ current recordings from excised macropatches and from whole cell performed on rat β-cells. These currents depend on the number of channels within the patch and on their open probability. At 0 μM Ca^2+^ with Cl^−^ as only permeant ion (symmetrical 150 mM NMDG-Cl [78]), no currents were observed (Fig. 5a on excised patches, Fig. 5n on whole cell). At 1 μM Ca^2+^, outwardly rectifying Cl^−^ currents were observed. The outward currents were composed of a small instantaneous time-independent component and a large slowly activating time-dependent component (Fig. 5b on single excised patches and 5o on whole cell). At 2 μM Ca^2+^, the main component of the current became instantaneous and time-independent. The inward and outward Cl^−^ currents reached steady-state quasi-instantaneously and became almost symmetrical so that the current–voltage relationship became approximately linear (Fig. 5c, f, g, i on single excised patches). Thus, in rat β-cells, Cl^−^ currents critically depend on [Ca^2+^]_i_ which is a characteristic of CaCCs [22, 39]. The permeability sequence of monovalent anions, which is also characteristic by CaCCs, was analyzed in whole-cell experiments replacing extracellular 150 mM NMDG-Cl by equimolar NMDG-NO_3_ or NMDG-Br. Pipette solution contained 150 mM NMDG-Cl and 1 μM Ca^2+^ (neither cyclic AMP (cAMP) nor ATP). An agar-KCl 3M salt bridge provided the junction between the Ag/AgCl reference electrode and the bath. Permeability ratios (P_x_/P_Cl_) of nitrate and bromide anions were calculated from the shifts of the reversal potentials using Goldman, Hodgkin, and Katz equation. The currents observed were outwardly rectifying. Replacing NMDG-Cl by NMDG-NO_3_ increased the current amplitude at positive potentials and shifted the reversal potentials toward negative potentials from −0.71 ± 0.91 to −16.65 ± 1.34 mV (Fig. 5j, k, top, *n* = 11, *P* = 8.33 × 10^−7^), i.e., by −15.94 ± 1.49 mV. Replacing NMDG-Cl by NMDG-Br had similar effects but shifted the reversal potentials toward less negative potentials from −0.38 ± 1.09 to −9.66 ± 1.51 mV (Fig. 5l, k, bottom, *n* = 9, *P* = 6.5 × 10^−5^), i.e., by −9.28 ± 1.23 mV. The relative permeabilities (P_x_/P_Cl_) of these anions were thus NO_3_^−^ (1.83 ± 0.10) > Br^−^ (1.42 ± 0.07) > Cl^−^ (1.0) (Fig. 5m) consistent with the values reported for endogenous CaCCs and Ano1-transfected cells [22, 39, 78]. We then investigated the effects of blocking Ano1 on the Cl^−^ currents. In these experiments, pipette and bath solutions contained 150 mM NMDG-Cl. On inside-out excised macropatches, 10 μM T-AO1 in the pipette solution preincubated for 5 min abolished 2 μM Ca^2+^-activated Cl^−^ currents at +100 mV (Fig. 5a, c, e, f; currents at 2 μM Ca^2+^: 226.10 ± 47.39 pA, *n* = 8; with T-AO1: 19.73 ± 4.32 pA, *n* = 12, *P* = 0.019 which is not different from current at 0 μM Ca^2+^: 6.80 ± 0.94 pA, *n* = 12, *P* = 0.071). Blocking Ano1 antibodies ab72984 1:100 [14] in the pipette solution (extracellular side of the patch) preincubated for 4 min totally abolished 2 μM Ca^2+^-activated Cl^−^ currents at +100 mV compared with boiled ab72984 1:100 in the pipette (Fig. 5a, g–i; control current at 2 μM Ca^2+^ with boiled antibody: 191.59 ± 17.75 pA, *n* = 18 vs. with active antibody: 15.44 ± 4.01 pA, *n* = 13, *P* < 0. 001 which is not different from current at 0 μM Ca^2+^, *P* > 0.999). Whole-cell current activated by 1 μM Ca^2+^ in the pipette was similarly strongly inhibited in the presence of 10 μM T-AO1 (bath) for 2 min (Fig. 5n–q; 1 μM Ca^2+^-activated control normalized current at +100 mV: 451.93 ± 52.71 pA/pF, *n* = 21; with T-AO1: 98.78 ± 15.31 pA/pF, *n* = 15, *P* = 0.001). In whole-cell experiments, ab72984 in the pipette (cytosolic side) always reduced 1 μM Ca^2+^-activated Cl^−^ currents at +100 mV measured at different times (Fig. 5r–t; boiled vs. active antibody at 6 min: 436.61 ± 29.03 pA/pF, *n* = 14 vs. 194.48 ± 29.03 pA/pF, *n* = 13, *P* = 1.26 × 10^−4^; at 8 min: 379.48 ± 34.72 pA/pF, *n* = 12 vs. 147.71 ± 16.01 pA/pF, *n* = 12, *P* = 4 × 10^−6^; at 10 min: 258.06 ± 25.37 pA/pF, *n* = 10 vs. 114.82 ± 10.69 pA/pF, *n* = 10, *P* = 2.15 × 10^−4^; at 12 min: 223.33 ± 45.20 pA/pF, *n* = 5 vs. 101.60 ± 10.05 pA/pF, *n* = 8, *P* = 0.007).

### Single-channel Cl^−^ currents from inside-out patches isolated from rat β-cells are consistent with Ano1 currents

**Fig. 6:**
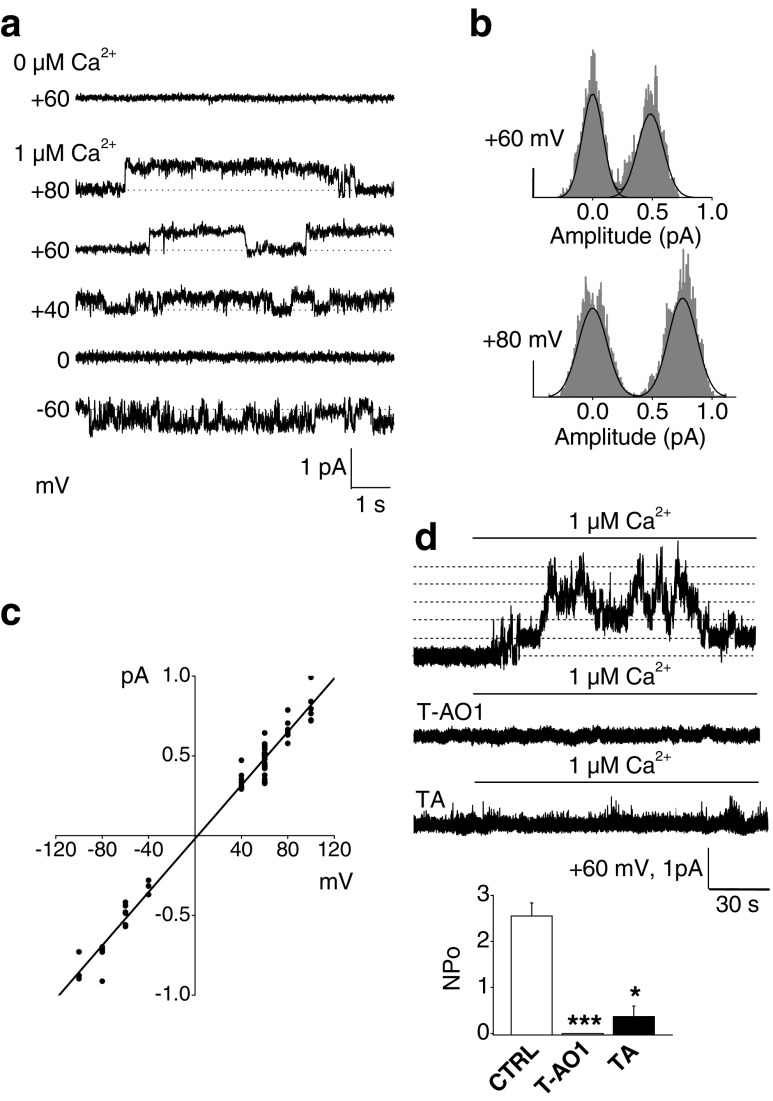
Single-channel Cl^−^ currents from inside-out patches excised from rat β-cells. Pipette and bath solutions contained 150 mM NMDG-Cl; pipette contained also 10 μM nifedipine and 10 μM glibenclamide. Sampling rate, 5 kHz; 1-kHz filter setting; 100-Hz final digital filtration. Filled pipette resistance, 20 MΩ. *Dotted lines* indicate zero-current or single-channel levels. **a** Representative recordings. Single-channel currents are activated by 1 μM Ca^2+^ in the bathing solution. **b** Representative number of events–amplitude histograms at +60 and +80 mV. Single-channel amplitudes were obtained from Gaussian fit. The *scale bars* indicate 250 events. **c** Current–voltage relationship of single-channel Cl^−^ currents activated by Ca^2+^. A single-channel conductance (*γ*) of 8.37 ± 0.15 pS was calculated from a linear fit (*r* = 0,989, *P* < 0.0001) on all the points (*n* = 65: +100 mV, *n* = 6; +80 mV, *n* = 7; +60 mV, *n* = 28; +40 mV, *n* = 7; −40 mV, *n* = 3; −60 mV, *n* = 6; −80 mV, *n* = 5; −100 mV, *n* = 3 experiments performed on six preparations of rat dispersed islet cells). **d** Time course of channel activity before and during exposure to 1 μM Ca^2+^ in the absence of inhibitors (*n* = 5), in the presence of T-AO1 100 μM (*n* = 7) and in the presence of TA 100 μM (*n* = 6) in the pipette solution at +60 mV. *Upper panel*: representative recordings in presence (or not) of the inhibitors. *Lower panel*: mean *NPo* (±SEM) values, i.e., the product of the number of channels in a patch (*N*) by the open probability (*Po*), calculated for 2 min, after 15-s stimulation with Ca^2+^. The *n* experiments were performed on two preparations of rat dispersed islet cells. Kruskal–Wallis test on **d**, *P* < 0.001, *P < 0.05, ***P < 0.001 vs. control (Mann–Whitney-type tests with Dunn–Bonferroni correction)

In the absence of Ca^2+^ in the bathing medium, no current was observed, while 1 μM Ca^2+^ activated single-channel Cl^−^ current (filled pipette resistance 20 MΩ). Representative single-channel Cl^−^ current recordings are shown in Fig. 6a; their amplitude was calculated by Gaussian fit to all-point histograms of recordings at a sampling rate of 5 kHz (Fig. 6b). The observed amplitudes of single-channel Cl^−^ currents were small, as also described for CaCCs by Nilius et al. [49, 50]. The single-channel current–voltage relationship (which plots the amplitude of one channel independently of its open probability vs. voltage) was linear (*r* = 0.989, *P* < 0.0001, Fig. 6c). Its slope, i.e., the single-channel conductance (*γ* = 8.37 ± 0.15 pS), exactly matches the conductance described for Ano1 in similar conditions in transfected Ano1/ET_A_R-HEK 293 cells [78]. The channel open probability was more important at positive voltages and decreased toward negative potentials, a well-known characteristic of CaCCs [49, 50], as confirmed by outwardly rectifying whole-cell and excised macropatch Cl^−^ currents activated by 1 μM Ca^2+^ (Fig. 5b, o). The effect of the inhibitors T-AO1 and TA, considered as specific for Ano1 [47, 48], was evaluated by measuring their effect on *NPo* at +60 mV (Fig. 6d). The inhibitors were added in the pipette solution before sealing. *NPo* was calculated for 2 min after 15-s stimulation with 1 μM Ca^2+^. For control patches, *NPo* was 2.55 ± 0.28 (*n* = 5). In presence of T-AO1, no opening was observed (*n* = 7, *P* < 0.001), and with TA, *NPo* drastically decreased to 0.36 ± 0.23 (*n* = 6, *P* = 0.0041). The abolishment of single-channel Cl^−^ current attests that Ano1 is indeed the target affected by these inhibitors. Recordings were validated only when patches showed one or a few single-channel events at the beginning of the measurements (immediately after excision, i.e., shortly after NaCl medium containing 1-mM Ca^2+^ replacement) or at + 100 mV at the end of recordings.

### Effect of Ano1 inhibitors on whole-cell Ba^2+^ currents from rat dispersed β-cells

**Fig. 7:**
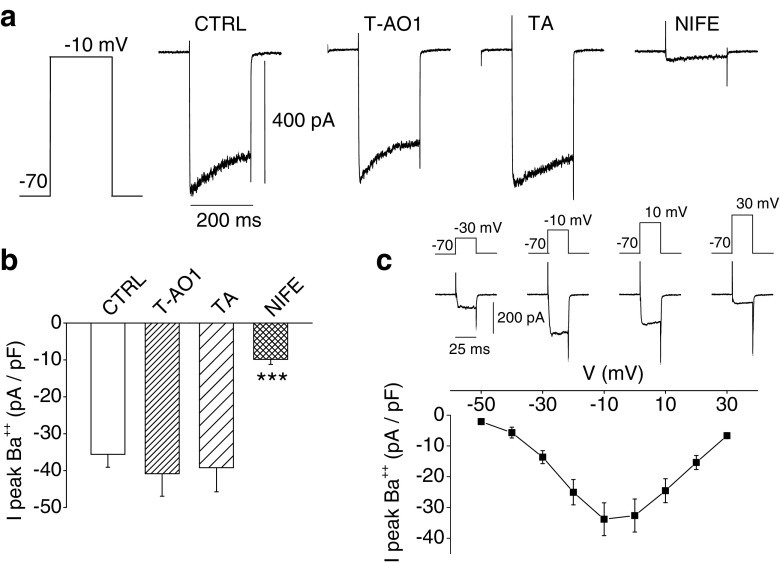
Whole-cell Ba^++^ currents from rat dispersed β-cells. The bathing solution contained the following (in mM): 75 BaCl_2_, 10 Hepes, 35 NMDG-gluconate (pH 7.3 with NMDG, osmolarity 310 mOsm/l) in presence (or not) of T-AO1 (100 μM), TA (100 μM), or nifedipine (10 μM). Pipette solution contained the following (in mM): 103 NMDG-Cl , 10 Hepes, 3 MgATP, 43 NMDG-gluconate, and 10^−4^ CaGluconate_2_ (pH 7.15 with NMDG, osmolarity 310 mOsm/l). Sampling rate, 5 kHz; 1-kHz filter setting. **a** Representative whole-cell currents being observed during 200-ms depolarization to −10 mV from a holding potential of −70 mV in the presence (or not) of T-AO1, TA, or nifedipine (NIFE) in the bathing medium. **b** Summary of normalized whole-cell peak Ba^2+^ currents observed with the same protocol as in **a**. For control experiments, *n* = 27. While T-AO1 (*n* = 17) and TA (*n* = 16) had no effect on the Ba^2+^ current, nifedipine potently reduced it (*n* = 17). **c** Normalized whole-cell peak inward Ba^2+^ current–voltage relation observed during 25-ms depolarization. Voltage steps were applied from a holding potential of −70 mV, *n* = 6. The current values were corrected for leakage and capacity currents. Experiments of these sets were carried out on two preparations of rat dispersed islet cells. Kruskal–Wallis test on **b**, *P* < 0.001, ***P < 0.001 vs. control (Mann–Whitney-type tests with Dunn–Bonferroni correction)

Although the current evidence points to a specific effect on Cl^−^ channels, it could still be argued that these inhibitors directly affect also the opening of L-type Ca^2+^ channels. Whole-cell Ba^2+^ current is representative of Ca^2+^ current mediated by L-type Ca^2+^ channels. Ba^2+^ currents were recorded during 200-ms depolarizations to −10 mV from a holding potential of −70 mV in the presence (or not) of T-AO1, TA, or nifedipine in the bathing medium (Fig. 7a). Note that a rundown of the currents occurred after a few stimulations [2]. Nifedipine largely inhibited the current (Fig. 7a, b). The appearance of the current (*I*)–voltage (*V*) relationship (Fig. 7c) together with the nifedipine inhibition thereby confirms the implication of L-type Ca^2+^ channels as mediators of these Ba^2+^ currents [2, 59, 60, 77]. Figure 7b summarizes the normalized whole-cell peak inward Ba^2+^ currents observed in the same conditions as in Fig. 7a. Control value averaged −35.60 ± 3.49 pA/pF (*n* = 27). Neither T-AO1 nor TA had any inhibitory effect on the Ba^2+^ current: T-AO1-exposed cells exhibited average current values of −40.84 ± 6.09 pA/pF (*n* = 17 cells, *P* > 0.5), and cells perfused in presence of TA, −39.21 ± 6.51 pA/pF (*n* = 16, *P* > 0.5). Nifedipine quite potently and significantly reduced the current to 27.7 ± 3.9 % of the average control value, i.e., −9.85 ± 1.37 pA/pF (*n* = 17, *P* < 0.001). The average cell capacitance was 7.66 ± 0.35 pF (*n* = 77).

### Effect of reduced intracellular Cl^−^ and HCO_3_^−^ on membrane potential oscillations in dispersed β-cells

**Fig. 8:**
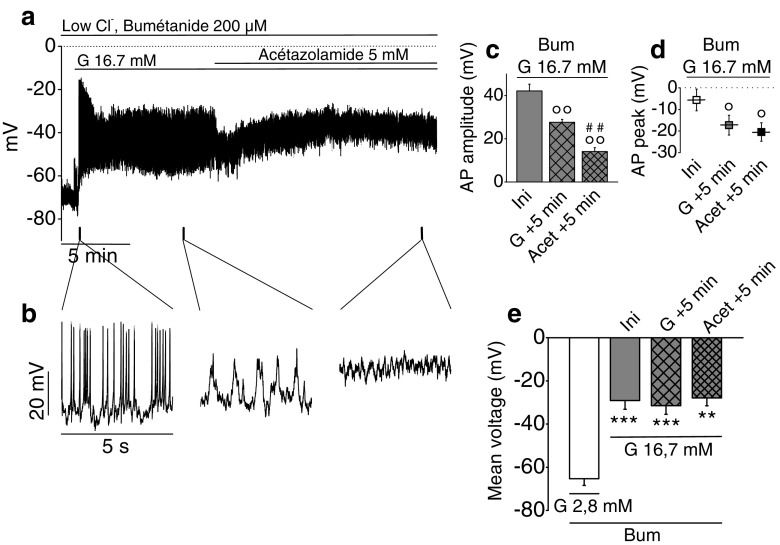
Effect of glucose on membrane potential oscillations in single rat β-cells exposed to low Cl^−^/HCO_3_^−^-free medium. Zero-current nystatin-perforated patch-clamp voltage recordings. Sampling rate, 18 kHz; 2-kHz filter setting. *Dotted lines* represent zero-voltage level. β-Cells were continuously exposed to 200 μM bumetanide (Bum), a NKCC1/2 inhibitor, and stimulated with 16.7 mM glucose (G 16.7 mM) in Hepes-buffered NaCl solution without bicarbonate containing 20 mM Cl^−^. After 10-min glucose stimulation, 5 mM acetazolamide (Acet), a carbonic anhydrase inhibitor, was added to further reduce intracellular HCO_3_^−^. **a** Representative recording. **b** Extended timescale of recording **a. c** AP amplitude at various times, i.e., at the beginning of the spiking activity before rundown (Ini), after 5 min in presence of glucose stimulation (G + 5 min), and after 5 min in presence of acetazolamide (Acet + 5 min). **d** AP peak voltage at similar times. **e** Mean voltage at similar times. For all results, *n* = 6 experiments performed on one preparation of rat islets. Repeated measures ANOVA test on **c**, *P* = 2.50 × 10^−5^; **d**, *P* = 1.29 × 10^−4^; and **e**, *P* = 7.42 × 10^−10^. **P < 0.01, ***P < 0.001 vs. 2.8-mM glucose condition in presence of bumetanide; °*P* < 0.05, °°*P* < 0.01 vs. Ini; ##*P* < 0.01 vs. G + 5 min (Sidak tests)

The last series of experiments aimed at investigating the possible role of Ano1 in the exit of bicarbonate anions from islet cells. To reduce intracellular Cl^−^ and HCO_3_^−^, we used here an incubation medium containing no HCO_3_^−^ and low Cl^−^ concentration (20 mM) with bumetanide (200 μM), the latter preventing Cl^−^ recycling through the Na^+^–K^+^–2 Cl^−^-cotransporters NKCC1/2 [1]. The addition of 16.7 mM glucose (Fig. 8a, b) elicited spiking activity whose AP amplitude reduced within 1 min from 42.17 ± 3.16 to 27.62 ± 1.37 mV; Δ = 14.55 ± 2.96 mV (Fig. 8c, *n* = 6, *P* = 0.00441). Concomitantly, AP peak repolarized from −5.58 ± 5.02 to −17.28 ± 4.59 mV; Δ = 11.70 ± 1.90 mV (Fig. 8d, *n* = 6, *P* = 0.00163). The latter parameters were further reduced upon addition of 5 mM acetazolamide: AP amplitude was decreased to 14.10 ± 1.80 mV, i.e., to 35.2 ± 6.1 % of the initial value (Fig. 8c, *n* = 6, *P* = 0.00123). AP peak repolarized to −20.45 ± 4.28 mV (Fig. 8d, *n* = 6, *P* = 0.00192). The resting potential averaged −65.29 ± 3.14 mV. During the first minute of spiking activity, the membrane voltage averaged −29.10 ± 4.04 mV (Fig. 8e, *P* = 2.92 × 10^−4^). The subsequent reductions in AP peak and amplitude observed including in the presence of acetazolamide did not significantly affect the average membrane potential.

## Discussion

The β-cell is an excitable cell in which raising glucose concentration above a threshold value of 5.5–6 mM induces a characteristic fluctuating electrical activity. This cyclic pattern alternates repetitive phases of depolarized plateau potential (slow waves) on top of which Ca^2+^-dependent AP superimposes (“active phase”) with partially repolarized silent phases [15–17, 31]. Variations in membrane potential are instrumental in inducing Ca^2+^ variations [5, 59, 61], hence insulin release. Importantly, insulin release is sustained only when these membrane potential fluctuations are also sustained [7, 24, 30]. Glucose also induces an increase in ^36^Cl^−^ efflux from mice islet β-cells. Severe reduction in this efflux by incubation in low Cl^−^ media inhibited the electrical bursting pattern together with the ^45^Ca^2+^ uptake and, therefore, the glucose-stimulated insulin secretion [65, 67]. This glucose-induced Cl^−^ efflux from rat pancreatic islet β-cells increases in proportion to the extracellular glucose concentration in the range of 6 to 20 mM [43].

The present experiments were undertaken to find out whether the observed Cl^−^ and possibly HCO_3_^−^ efflux from rat pancreatic islet β-cells are mediated by Ano1. As a first approach, we demonstrated the expression of Ano1 in rat pancreatic islet β-cells by RT-PCR (including sequence analysis), immunohistochemistry (including inhibition of labeling by Ano1 synthetic peptide), and western blotting (Fig. 1). Of interest, transcripts of the expected size of Ano1 were also found in preparations from human pancreatic tissue, thus suggesting that Ano1 is also expressed in human. As a second approach, we measured the effect of Ano1 inhibitors on GSIS. Exposure to 100 μM T-AO1 totally prevented the increment of insulin secretion induced by 8.3 and 16.7 mM glucose; exposure to TA was qualitatively similar with a complete inhibition at 8.3 mM glucose but not total (67 %) at 16.7 mM glucose. The concentration of inhibitors necessary to abolish GSIS was admittedly rather high, but this is probably merely related to diffusion into the center of islet where β-cells reside [33]. We therefore also looked at the effect of the blocking Ano1 antibodies ab72984 [14] which at 1:100 abolished 16.7 mM GSIS (Fig. 2 (c3)).

In β-cell, glucose induces a typical pattern of cyclic fluctuations of the membrane potential. Yet, while the rhythmic electrical activity of the heart evidenced in the electrocardiogram can be adequated to the gating of a series of well-identified ionic channels [4], only a few channels have been well identified as critically involved and necessary for the glucose-induced fluctuations of the β-cell membrane potential. Glucose is absolutely necessary to induce these cyclic fluctuations as, when present below its threshold, the membrane potential remains quite stable at around −70 mV. Three main phases can be individualized: (1) a slow depolarization ascribed to the closure of K_ATP_ channels reproduced by sulfonylurea drugs and inhibited by diazoxide, (2) cyclic waves of depolarization interrupted by silent phases of partial repolarization whose length decreases when glucose concentration raises, and (3) AP ascribed to the opening of L-type Ca^2+^ channels, inhibited by nifedipine. The last two phases are reminiscent of the pacing activity of interstitial cells of Cajal in the intestine [35, 79] except that glucose is absolutely required to induce this pacing activity in β-cell. A third objective of this study was to show that Ano1 mediates the cyclic waves of depolarization, which are absolutely necessary to reach the threshold of the L-type Ca^2+^ channels (triggering AP). These waves of depolarization can be at least partially attributed to the gating of Ano1 (with Cl^−^ exit from the β-cell), as exposure to T-AO1 or TA inhibitors almost totally suppressed AP or at least drastically reduced AP rate. The more potent T-AO1 partially repolarized the β-cell (Figs. 3 and 4). Drastic reduction in AP rate severely impairs insulin secretion (Figs. 2, 3, and 4). Furthermore, the inhibitors repolarized the AP peak which also plays a role in Ca^2+^ influx. Finally, TA reduced by one-third AP amplitude in mice β-cells from whole islet upon 16.7-mM glucose stimulation, while no more AP was observed in rat dispersed β-cells. A contrario, increased AP rate is known to raise Ca^2+^_i_ and insulin secretion by SK4^−/−^ mice [19]. Increased AP amplitude (with increased AP peak) observed by blocking large-conductance Ca^2+^-activated K^+^ channel current (I_BK_) with paxillin significantly increased Ca^2+^ entry and insulin secretion [34], while reduction in AP amplitude by tetrodotoxin drastically reduced the stimulation of insulin secretion in human β-cells [10].

The presence of active Ano1 channels in β-cells was demonstrated in inside-out excised patches and in conventional whole-cell patch-clamp experiments on dispersed cells. Chloride currents from rat β-cells display typical properties of CaCCs (Fig. 5). No Cl^−^ current was observed in the absence of intracellular Ca^2+^ (symmetrical 150 mM NMDG-Cl condition in the absence of cAMP and ATP). One-micromolar Ca^2+^ directly triggered strongly outwardly rectifying currents showing a large slowly activating time-dependent component, and this behavior completely changed to nearly linear at 2 μM [Ca^2+^]_i_ with a large instantaneous, time-independent component. This different behavior depending on [Ca^2+^]_i_ is characteristic by CaCCs [22, 39] now identified as Ano1 [64]. The permeability sequence of monovalent anions, which is also characteristic by CaCCs, was analyzed in whole-cell experiments (1 μM [Ca^2+^]_i_) replacing extracellular 150 mM NMDG-Cl by equimolar NMDG-NO_3_ or NMDG-Br. Permeability ratios (P_x_/P_Cl_) of nitrate and bromide anions calculated from the shifts of the reversal potentials using Goldman, Hodgkin, and Katz equation were NO_3_^−^ (1.83 ± 0.10) > Br^−^ (1.42 ± 0.07) > Cl^−^ (1.0), consistent with those reported for endogenous CaCCs and Ano1-transfected cells [22, 39, 78]. The observed currents were outwardly rectifying, excluding cystic fibrosis transmembrane conductance Regulator (CFTR) currents. Furthermore, blocking Ano1 antibodies ab72984 [14] directed against the fifth and sixth transmembrane domains that constitute the pore-forming region and T-AO1 used at 10 μM here directly in contact with the cell membrane completely abolished the 2 μM Ca^2+^-activated Cl^−^ currents from inside-out excised macropatches from rat β-cell. A similar inhibitory effect of T-AO1 on 1 μM Ca^2+^-activated Cl^−^ currents was observed in whole-cell experiments. These observations prove that active Ano1 channels are present in the β-cell membrane. Finally, single-channel Cl^−^ currents were examined in inside-out excised patches from rat β-cell membrane (Fig. 6). The activation of the channels was Ca^2+^-dependent, as in the absence of Ca^2+^ in the bath (cytoplasmic side), no current was observed. At 1 μM Ca^2+^, a unique Cl^−^ conductance was evoked. The channel involved exhibited a linear *I*/*V* relationship, with a small slope conductance of 8.37 ± 0.15 pS matching the Ano1 conductance reported under similar conditions (symmetrical 150 mM NMDG-Cl) [55, 78]. Its opening (measured by *NPo*) was abolished in the presence of Ano1 inhibitors (T-AO1 or TA) [47, 48]. Again, these single-channel experiments were performed in absence of ATP, PKA, and cAMP and channel activation required Ca^2+^, excluding CFTR [11, 20] under these conditions although it has a nearly similar conductance (10 pS).

L-type Ca^2+^ channels were not affected by Ano1 inhibitors, as shown by the whole-cell Ba^2+^current experiments (Fig. 7), thus ruling out unspecific effect of the inhibitors on Ca^2+^ channels.

Interestingly, the direct cause of Ano1 opening is still in debate. Potential candidates are (i) an increase in intracellular Ca^2+^, an enhanced Ano1-Ca^2+^ sensitivity induced by plasma membrane depolarization, or still Ca^2+^–calmodulin dependence; the Ca^2+^ increase could be very localized in microdomains of excluded cytoplasmic volume and defined by the close association between restricted plasma membrane domain and the endoplasmic reticulum (ER) [36, 73], as Ano1 seems to tether the plasma membrane to the ER as shown in the interstitial cells of Cajal [80]; (ii) an ATP-dependent mechanism (as for example, phosphorylation, dephosphorylation) [55] or an unknown dependence on cytosolic ATP [73]; (iii) a dependence of Ano1 opening from the CFTR as Edlund et al. [21] suggested a direct interaction between Ano1 and the CFTR when the latter is open by the cAMP/PKA pathway. However, the expression level of Ano1 is far greater than that of the CFTR. Also, their experiments were all performed in the presence of forskolin or GLP-1, which was not the case in the present study. Finally, these authors hypothesized that CFTR/Ano1 mediates a Cl^−^ influx during AP, i.e., when the Cl^−^ electrochemical potential favors Cl^−^ into the cytosol and eventually into the granules (via ClC-3 channel). In another setting, Kunzelmann and Mehta [37] also suggested that the CFTR may play a role in Ano1 opening. Although the link between CFTR and Ano1 remains to be defined, this suggestion is quite interesting as another recent study strongly implicated the CFTR chloride channel in the glucose-induced β-cell plasma membrane depolarization [26]. Thus, besides Ano1, the CFTR may also participate to the Cl^−^ efflux current upon glucose stimulation as the latter increases cAMP production [58]. However, diabetes observed in cystic fibrosis is rather uncommon before the first decade [46], suggesting that CFTR is not of absolute necessity for GSIS but could represent a way to open Ano1 under some physiological conditions. Clearly, the question of how glucose triggers Ano1 opening is not solved, and among attractive useful candidates, some of the numerous TRP channels expressed in the rat β-cell [13] could also be raised as they depolarize the cell and often mediate Ca^2+^ influx. For instance, an increment of reactive oxygen species (ROS) production, as observed upon 16.7-mM glucose stimulation of mouse or rat β-cells islets [41, 57], activated TRPM2 and increased [Ca^2+^]_i_ in INS-1E and human β-cells [3]. Furthermore, insulin secretion was impaired in TRPM2^−/−^ mice [74]. In addition, a decrease in glucose-induced Ca^2+^ oscillations responsible for a drastic reduction in insulin secretion was described in TRPM5^−/−^ mice [12].

As a final objective, we examined whether HCO_3_^−^ can also exit through Ano1. Bicarbonate ion appears to play a role in insulin secretion as (1) its omission from the extracellular medium drastically reduces insulin secretion [29]; (2) its entry into the β-cell appears mediated by NBCe1-B [27, 53]. This electrogenic Na^+^–HCO_3_^−^-cotransporter, expressed in rat and human islet β-cells [27, 71], carries the entry of one negative charge (stoichiometry: 1 Na^+^/2 HCO_3_^−^). Thus, HCO_3_^−^ entry is facilitated when the cell depolarizes and also when cytoplasmic Cl^−^ decreases, given that NBCe1-B has Cl^−^-binding motifs [69]; (3) its concentration depends on its production from glucose metabolism which generates H_2_O and CO_2_, partially converted into HCO_3_^−^ and H^+^ ions in the presence of mitochondrial type V carbonic anhydrase [68]. While the protons can probably recycle into the mitochondrial matrix or rapidly exit the β-cell through the sodium–proton exchanger NHE1, HCO_3_^−^ first accumulates within the cytoplasm; hence, it contributes to its alkalinization [18, 72] and possibly to cell volume increase. Acetazolamide-induced inhibition of carbonic anhydrase also decreases insulin secretion in rat pancreatic islets [54, 68]. Thus, upon glucose stimulation, intracellular HCO_3_^−^ appears to increase from two different sources: metabolically generated from mitochondrial pyruvate oxidation and absorbed from the extracellular medium through NBCe1-B. When cytoplasmic Cl^−^ is largely decreased as a result of its exit through Ano1, HCO_3_^−^ which has been accumulated within the cytoplasm can also exit through Ano1, as this channel has been shown to be permeant to HCO_3_^−^ [55]. When intracellular Cl^−^ was decreased, in the presence of bumetanide and 16.7 mM glucose, AP amplitude was reduced with repolarized AP peak. In these conditions, bumetanide prevented Cl^−^ recycling; hence, Ano1 current mediated the exit of HCO_3_^−^. Acetazolamide which partially reduces intracellular HCO_3_^−^ concentration decreased further AP amplitude and peak, in HCO_3_^−^-free media (Fig. 8). As shown by Sener et al. [68], acetazolamide also decreased GSIS in rat islets. Thus, glucose also appears to induce HCO_3_^−^ exit through Ano1.

Ano1^−/−^ mice do not survive more than a few days unlike Ano1^+/−^ mice which showed impaired insulin secretion [76]. This elegant study also completely supports a role for Ano1 in insulin secretion. The authors reported that insulin and Ano1 genes lie on the same human chromosome 11, that they interact with each other, and that this interaction is strengthened, upon glucose stimulation. Twenty-five-millimolar glucose induced a threefold increased expression of Ano1 after 1 h, whereas its expression was decreased in the presence of siRNA against the insulin promoter.

Thus, the glucose-induced gating of Ano1 may be as important as the K^+^_ATP_ channel closure to induce β-cell depolarization. We further suggest that Ano1 cyclic gating is responsible for the oscillatory pattern of membrane potential upon glucose stimulation with alternating active phases with AP and partially repolarized silent phases. These are instrumental in generating Ca^2+^_i_ oscillations and sustained insulin secretion. Blocking Ano1 nearly completely abolished the membrane potential oscillations as well as GSIS.

In conclusion, the present results demonstrate that in murine β-cells, glucose-induced opening of Ano1 is absolutely critical to reach the threshold potential for opening the L-type Ca^2+^ channels.
